# Data from three camera trapping pilots in the Amsterdam Water Supply Dunes of the Netherlands

**DOI:** 10.1016/j.dib.2024.110544

**Published:** 2024-05-21

**Authors:** Julian C. Evans, Rotem Zilber, W. Daniel Kissling

**Affiliations:** Institute for Biodiversity and Ecosystem Dynamics (IBED), University of Amsterdam, P.O. Box 94240, 1090 GE Amsterdam, the Netherlands

**Keywords:** Biodiversity monitoring, Digital sensors, Dune ecosystem, Exclosures, FAIR, Mammals, Protected areas, Species identification algorithms

## Abstract

This paper presents the data (images, observations, metadata) of three different deployments of camera traps in the Amsterdam Water Supply Dunes, a Natura 2000 nature reserve in the coastal dunes of the Netherlands. The pilots were aimed at determining how different types of camera deployment (e.g. regular vs. wide lens, various heights, inside/outside exclosures) might influence species detections, and how to deploy autonomous wildlife monitoring networks. Two pilots were conducted in herbivore exclosures and mainly detected European rabbits (*Oryctolagus cuniculus*) and red fox (*Vulpes vulpes*). The third pilot was conducted outside exclosures, with the European fallow deer (*Dama dama*) being most prevalent. Across all three pilots, a total of 47,597 images were annotated using the Agouti platform. All annotations were verified and quality-checked by a human expert. A total of 2,779 observations of 20 different species (including humans) were observed using 11 wildlife cameras during 2021–2023. The raw image files (excluding humans), image metadata, deployment metadata and observations from each pilot are shared using the Camtrap DP open standard and the extended data publishing capabilities of GBIF to increase the findability, accessibility, interoperability, and reusability of this data. The data are freely available and can be used for developing artificial intelligence (AI) algorithms that automatically detect and identify species from wildlife camera images.

Specifications Table

**Table:** 

Subject	Systematics, Ecology and Behavior
Specific subject area	Development of automated biodiversity monitoring methods using wildlife cameras and artificial intelligence (AI) algorithms for digital species identification.
Type of data	Image, Raw, Processed
Data collection	Images were recorded with two camera trap models (Snyper Commander 4G Wireless, Wilsus Tradenda 4G Wireless) which were operated autonomously with 12 V/2A solar panels and transmit data automatically via 4G. Cameras are triggered by a passive infrared (PIR) sensor and use an infrared flash at night. They were run with either a regular (52°) or wide lens (100°) at 30, 40, or 50 cm height, and deployed over 42, 109 and 747 days, respectively. They recorded 5 images per trigger (multi-shot), each with an image size of 5 megapixels. Species identifications were made by a combination of AI and humans using the Agouti platform (https://www.agouti.eu), with all identifications being verified by human experts.
Data source location	Data were collected in the Amsterdam Water Supply Dunes of the Netherlands (52.34692764009705, 4.5275671675946105). The pilots were conducted in different parts of this nature reserve: Pilot 1: exclosures Zilkerpad, van Limburgstirum Vallei and Wolfsveld Pilot 2: exclosure Zeeveld Noord Pilot 3: outside exclosures in area Westweg
Data accessibility	All data are available except for the raw images containing humans, which have been excluded. The observations derived from these images (i.e. presence records of humans) are still included in the data package. Repository name: Repository of camera trap data recorded during three pilot studies of the Amsterdamse Waterleidingduinen Data identification number: 10.5281/zenodo.10671148 Direct URL to data: https://zenodo.org/records/10671148

## 1 Value of the Data

• Camera traps have emerged as a powerful, non-invasive and cost-efficient tool for studying the abundance, diversity and distribution of animals, especially of medium-to-large ground-living mammal and bird species [[1], [2], [3]]. Camera traps are often deployed with short- and medium-term deployments (< 4 weeks) because they require manual replacement of batteries and manual retrieval of SD cards. Recent technological advances have resulted in wildlife cameras that can be operated autonomously (with solar panels) and with functionalities for automated data transmission (e.g. 4G). This allows the collection of continuous 24/7 time series over extended time periods (e.g. >1 month up to several years) and can provide detailed insights into the habitat use, activity and occupancy of wildlife species. Continuous time series also allow identification of the optimal time window to obtain precise estimates of species richness and species detection rates, e.g. by splitting continuous camera deployments into shorter time intervals [4].
• It is important to conduct pilot studies before implementing a camera trap survey [4]. Pilot studies can help to refine the survey design and field protocols and show at which rates data accumulate. They can also provide crucial insights into the detectability of species. For instance, the camera height can have an impact on the number of detections obtained [1]. Pilot studies with different camera heights (e.g. between 20 and 50 cm) can therefore inform about the best way of deployment in a given habitat [4]. Cameras can also be purchased with different lens types, e.g. a regular lens (field of view 52°) or a wide lens (100°), and species detections might vary with different lens types. Moreover, testing cameras at locations with different habitats or with different experimental settings (e.g. inside/outside herbivore exclosures in rewilding projects) can help to estimate rates of data accumulation and data transmission.
• The processing of the vast amount of data that camera traps quickly generate is often a bottleneck in camera trap research. Supervised machine learning models, convolutional neural networks (CNNs) and other artificial intelligence (AI) algorithms are therefore increasingly developed to detect and classify species from camera trap images and to efficiently filter empty images [5]. However, such algorithms require labeled images for model training which are often not openly available. Hence, publishing free and open, well-annotated datasets (e.g. high-quality labels created by human experts) will allow AI algorithms for digital species identification to improve in robustness and accuracy [[5], [6], [7]]. The provided dataset is therefore not only providing free access to the derived species observations, but also to the manually classified images which can be used for training new machine learning models.

## 2 Background

The pilot studies were conducted to test the autonomous deployment of wireless 4G wildlife cameras with solar panels and automated data transmission in the coastal dunes of the Amsterdam Water Supply Dunes, The Netherlands. Monitoring and management of grazing mammals such as the European rabbit (*Oryctolagus cuniculus*) and the European fallow deer (*Dama dama*) is of key interest in this nature reserve, as they slow down the rate of natural succession and alter plant species composition and vegetation structure through grazing and digging. These grazers as well as predators such as the red fox (*Vulpes vulpes*) have typically been monitored using traditional survey methods such as transect and areal counts, but only once or twice a year. The study was aimed to test the feasibility and methodology of running long-term autonomous monitoring networks with wildlife cameras. This involved the testing of power usage, data transmission, and data accumulation, and the robustness of cameras to herbivore damage. Furthermore, the pilots specifically tested how the detection of focal species (rabbits, deer, foxes) differs with deployment heights, camera lens types and the placement in different habitats. The pilot also provides labelled images for the development of deep learning algorithms to automatically identify species.

## 3 Data Description

The repository [8] contains data stored in camera trap data package (Camtrap DP) format [9] for each of the three pilots. Camtrap DP is an open standard for the exchange and archiving of camera trap data using a standardized data structure [9]. Each data package consists of the following resources:

• **datapackage.json:** Contains metadata about the data package and camera trap project from which the data originates. Describes taxonomic, temporal, and spatial details.
• **deployments.csv:** Table of individual camera trap deployments, detailing exact location and times active of each camera deployment.
• **media.csv:** Table detailing every image in the data package. Lists the filenames and paths of images within the data package.
• **observations.csv:** Table of observations of species (or lack thereof) derived from image sequences.
• **events.csv:** Table linking observation events to media.
• **media folder:** Folder containing a subfolder for each deployment, which contains the raw images from that deployment.

Some additional notes, specific to these datasets:

• The deployment table contains “deployment tags”, which specify extra information about the deployment, formatted as key:value pairs, separated by pipes (‘|’). Of particular interest for these datasets are the tags that state lens angle, specify habitat type and identify paired cameras (e.g. to assess differences in species detections between cameras with regular and wide lens, respectively).
• In all three pilots, most observations are linked to sequences of images recorded within 120 s of each other. Hence, observations in these datasets are generally linked to an “event” (i.e. a sequence of images) rather than to an individual media file. This is noted in the ‘observationLevel’ field of the ‘datapackage’ JSON file and indicated on each row of the ‘observations’ table in the ‘observationLevel’ column. In order to make it easier for researchers to find the images that make up an observation we have added an ‘events’ table to link observation events and the media items that make up that event. This is an extension of the camera trap DP standard. By joining rows from the ‘observations’ table to the ‘event’ table based on the ‘eventID’ column, then joining rows from the ‘media’ table to this new table using the ‘mediaID’ column, per media item observations can be generated. Care should be taken when doing this however (see limitations section).
• All annotations were verified and checked by a human expert, even in cases where an observation is listed as being made by an AI algorithm.
• Whether or not an image is included in the data package is indicated by the ‘filePublic’ column in the media table. All raw images are included except for those where humans were detected. Images in which humans were detected have a ‘filePublic’ value of FALSE. Although the current location of these files within the Agouti platform (https://www.agouti.eu/) is recorded in the ‘filePath’ column, these files cannot be accessed. The ‘fileName’ of these filles is the original filename they possessed when uploaded to Agouti.
• Where ‘filePublic’ is TRUE, the `filePath` given is relative to the root of the data package (e.g. ‘media/<deployment>’) and the `fileName` of the file is the current name of the file within the data package (‘<mediaID>.JPG’). The full path of a media file within the data package can therefore be obtained by combining the ‘filePath’ and ‘fileName’ columns, in rows where `filePublic` is TRUE.

More details about individual metadata fields in the Camtrap DP format can be found on https://camtrap-dp.tdwg.org/.

## 4 Experimental Design, Materials and Methods

### 4.1 Camera trap overview

The first two pilots utilised the Snyper Commander 4 G Wireless camera (https://www.wildlifemonitoringsolutions.com/snyper-commander-4g-wireless), while the third pilot also used Wilsus Tradenda 4 G Wireless camera (https://www.wildlifemonitoringsolutions.com/wilsus-tradenda-4g-wireless). Both Synper and Wilsus cameras are very similar camera models and can be purchased with either a regular lens (field of view 52° and 60°, respectively) or a wide lens (both 100°). These cameras can be run autonomously for long time periods with a 12 V/2A solar panel and transmit images (and a daily report text file) automatically via 4 G (using MMS, email, or directly to an FTP server). The cameras are triggered by a passive infrared (PIR) sensor and use an infrared flash at night. In the pilot studies, the PIR sensor was set to medium sensitivity, as this seemed to be a good compromise between false triggers and no triggers During the studies, regular lens Snyper cameras used firmware version 4MR3WLrBB39 while the wide lens version used 4MR3WLwBB3. The Wilsus cameras (all wide lens) used firmware version 5MR3WLwC639.

In all deployments, cameras were set to record 5 images per trigger (multi-shot), each with an image size of 5 megapixels. Images in a trigger sequence are separated by 0.4 s. Images were either downloaded manually from the SD card of the camera or were automatically uploaded to an FTP server and then downloaded.

### 4.2 Annotation overview

Images from the pilots were classified in sequences, with each sequence consisting of images recorded within 120 s of each other. Image sequences were annotated using the Agouti platform (https://www.agouti.eu) [10]. We first applied available AI algorithms in Agouti to obtain species identifications for all image sequences. Images annotated between August 2021 until June 2022 used the ‘Western Europe species model Version 1’ while all images annotated between July 2022 until December 2022 used the ‘Western Europe species model Version 2’. All images annotated in 2023 used the ‘Western Europe species model Version 4a’. Date of annotation and the algorithm initially used can be found in the ‘observations’ table.

After the initial classification by AI, each image sequence was manually checked by a human (ecologist). Correct AI annotations were kept, wrong annotations were corrected, and image sequences which the AI algorithm was unable to classify (i.e. high uncertainty) were manually annotated. Uncertain identifications were sent to external experts for validation.

### 4.3 Pilot details

**Fig. 1:**
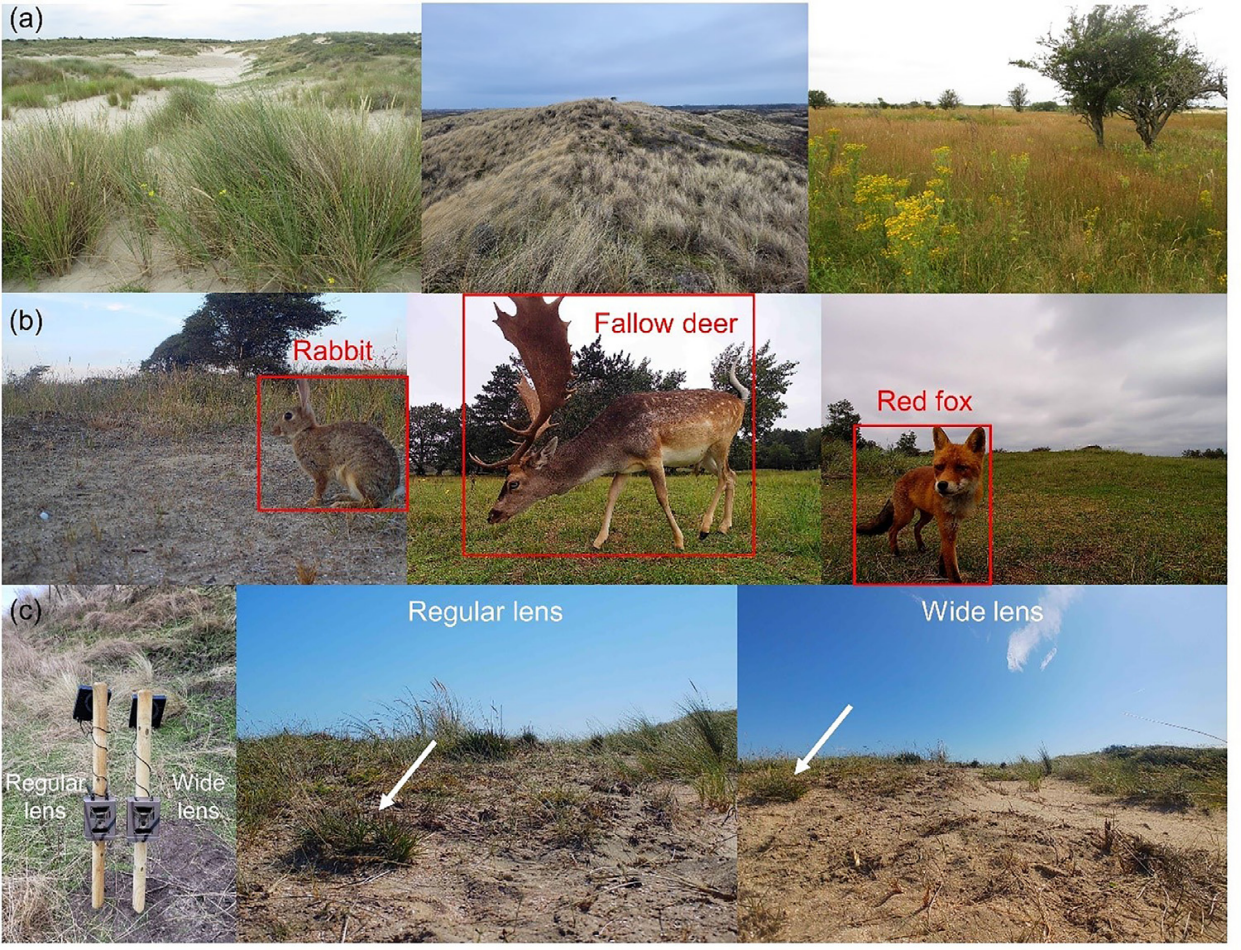
Camera trapping pilots in the Amsterdam Water Supply Dunes of the Netherlands. (a) Examples of coastal habitat with sand dunes (left, middle) and grassland in herbivore exclosure (right). (b) Focal wildlife species of interest to nature management include the European rabbit (*Oryctolagus cuniculus*), European fallow deer (*Dama dama*) and red fox (*Vulpes vulpes*). (c) Example of paired sampling design testing species detections with regular lens (field of view 52°) or a wide lens (100°) wildlife cameras.

Three pilots were conducted in the Dutch coastal dunes to test the monitoring of focal wildlife species and different lens angles of camera traps (Fig. 1). The first two pilots were conducted in exclosures (three exclosures in pilot 1, one exclosure in pilot 2) with a focus on rabbits and red fox. The third pilot was conducted at three locations outside exclosures, with a main focus on the European fallow deer.

#### 4.3.1 Pilot 1

Pilot 1 was primarily focused on testing camera deployment, data accumulation and data transmission (4 G coverage) in a remote location. Three Snyper Commander cameras were deployed from August 13th 2021 to August 2023 (approx. 747 days). The three cameras were placed within exclosures (locations Zilkerpad, van Limburgstirum Vallei and Wolfsveld in the Amsterdam Water Supply Dunes), at 30 cm above the ground. All images from 14th of August 2021 until 31st of December 2022 were annotated, with a total of 30,464 images over 505 days from the three cameras.

#### 4.3.2 Pilot 2

Pilot 2 was an initial test of the difference in species detection and data accumulation between a Snyper Commander camera with a regular lens (52°) and one with a wide lens (100°). The cameras were deployed at 30 cm above the ground within the exclosure Zeeveld Noord in the Amsterdam Water Supply Dunes from 14th of August 2021 to 24th of September 2021. During this pilot, a solar panel failure caused the cameras to stop recording data from the 24th of August 2021 to the 6th of September (14 days). During annotation, only days in which both cameras were operational were annotated. This led to a total of 1113 images over 28 days from the two cameras.

#### 4.3.3 Pilot 3

Pilot 3 was a further test of the effect of lens angle, as well as a test of the influence of camera height and being deployed outside exclosures (security, herbivore damage, more varied habitat types). At each of three locations (all in the area Westweg of the Amsterdam Water Supply Dunes), both a regular lens Snyper Commander camera (52°) and a wide lens Wilsus Tradenda camera (100°) were deployed (*n* = 6 cameras). The cameras were placed at different heights at each location (30 cm, 40 cm and 50 cm above the ground). A month of data from this pilot was annotated, from 1st March 2023 to 31st of March 2023. This led to a total of 16,020 annotated images over 31 days from six cameras.

## Limitations

The recording time listed in the exif metadata of the image files (and therefore in the media table) have some slight delays when compared to the timestamp printed on the image itself. Similarly, the exif timestamps of individual media items within a sequence run backwards compared to the actual times of individual images. If working with individual images or if exact times are required, the timestamps printed on the images could be extracted and used.

Each observation is linked to a sequence of images rather than to an individual image. While we provide the means of converting these event-level observations to per media observations by using the event table (as described above in the ‘Data description’ section), this might lead to some inaccurate labelling of individual images. For example, if an animal is only present in a portion of the images of a sequence, a naïve conversion to media level observations would result in incorrectly labelling empty images as having an animal present. Caution should therefore be taken to avoid false positives, perhaps by first running images through a simple object detection algorithm.

## Ethics Statement

The authors have read and follow the ethical requirements for publication in Data in Brief and confirming that the current work does not involve human subjects, animal experiments, or any data collected from social media platforms. Raw images of humans have been removed from the dataset.

## CRediT authorship contribution statement

**Julian C. Evans:** Software, Data curation, Writing – original draft, Writing – review & editing. **Rotem Zilber:** Conceptualization, Project administration, Methodology, Investigation, Writing – review & editing. **W. Daniel Kissling:** Funding acquisition, Project administration, Conceptualization, Supervision, Writing – original draft, Writing – review & editing.

## Data Availability

Repository of camera trap data recorded during three pilot studies of the Amsterdamse Waterleidingduinen (Original data) (Zenodo).
